# Strong Tribocatalytic Degradation of Organic Pollutants by Natural Shell Particles

**DOI:** 10.3390/nano16030194

**Published:** 2026-01-30

**Authors:** Yuqin Xie, Mingzhang Zhu, Zhenming Xu, Lina Bing, Wanping Chen, Zhenjiang Shen

**Affiliations:** 1 College of Physics and Electronic Engineering, Hainan Normal University, Haikou 571158, China; 202312070200012@hainnu.edu.cn (Y.X.); 202412070200020@hainnu.edu.cn (M.Z.); 202512070200018@hainnu.edu.cn (Z.X.); 410901@hainnu.edu.cn (L.B.); 2 School of Physics and Technology, Wuhan University, Wuhan 430072, China

**Keywords:** natural shell particles, tribocatalysis, dye degradation, high-concentration RhB solutions

## Abstract

This study presents a waste-valorization strategy by developing calcined natural shell particles (CNSP) derived from waste oyster shells as an efficient tribocatalyst for degrading high-concentration organic pollutants, a challenge for which conventional photocatalytic approaches are hindered by light shielding. The CNSP catalyst, confirmed as calcite CaCO_3_ with low surface area and stable crystalline structure, demonstrated exceptional efficacy in degrading Rhodamine B (RhB) solutions across a wide concentration range (50–300 mg/L) under mechanical friction, achieving 99% removal of 50 mg/L RhB in 1 h and 300 mg/L RhB in 18 h with a 0.5 g catalyst. This catalyst maintained a degradation efficiency of over 95% in a continuous six-cycle process. Mechanistic studies revealed that the tribocatalytic process generates reactive oxygen species (ROS), primarily hydroxyl (•OH) and superoxide ($•{O_{2}}^{-}$) radicals, which drive the decomposition of dye molecules. Electron paramagnetic resonance (EPR) spectroscopy directly confirmed the generation of these radicals. These findings establish CNSP as a promising, low-cost, and environmentally benign catalyst for wastewater treatment. This work not only provides a novel strategy for high-concentration dye removal but also reduces the environmental burden of aquaculture shell disposal. Further work is needed to evaluate its performance in real industrial effluents and with mixed pollutants.

## 1. Introduction

In 2024, China’s shellfish production reached 18.088 million tons, representing a year-on-year increase of 5.54% and accounting for over 80% of the global output. The rapidly expanding shellfish aquaculture industry in China consequently generates large quantities of shell waste, which, if improperly managed, can lead to environmental issues such as land occupation, odor emission, and ecological degradation [1]. Meanwhile, high-concentration organic dyes discharged from textile, printing, and pharmaceutical industries pose severe threats to aquatic ecosystems and human health due to their persistence, toxicity, and potential carcinogenicity [2,3,4]. Addressing these two pressing environmental challenges requires innovative strategies that align with the principles of a circular economy.

Waste shells, mainly composed of calcium carbonate (CaCO_3_), have been widely valorized in environmental and material-related applications, which can be broadly classified into structural/bioceramic uses and adsorptive or catalytic systems. For structural and bioceramic applications, waste shells are directly utilized as raw materials for composites or bioceramic scaffolds, owing to their favorable mechanical properties and biocompatibility [5,6,7]. However, these applications do not directly address the degradation of organic pollutants. In adsorptive systems, shell-derived materials are chemically converted into calcium-based adsorbents for the removal of heavy metals or dyes [8,9,10]. Despite their low cost and sustainability, their adsorption capacity is generally limited, particularly in high-concentration wastewater. In addition, advanced oxidation processes (AOPs) are regarded as green alternatives for pollutant degradation, primarily through the in situ generation of reactive oxygen species (ROS, e.g., •OH and $•{O_{2}}^{-}$) [11,12]. Among them, photocatalysis utilizes light energy to excite semiconductor catalysts (e.g., TiO_2_), initiating redox reactions [13]. However, industrial dyeing and printing processes often generate high-concentration wastewater (up to hundreds of milligrams per liter), where the severe light-shielding effect drastically reduces photocatalytic efficiency and limits its practical application. In contrast, the emerging mechano-driven AOP of tribocatalysis utilizes mechanical energy rather than light as the driving force for ROS generation, making it inherently less sensitive to light-shielding effects. This feature suggests that tribocatalysis is not merely an alternative to photocatalysis, but a complementary approach particularly suited for treating highly concentrated and optically opaque dye wastewaters.

Tribocatalysis, an emerging mechanical energy-driven technology, offers a promising solution to this limitation. It induces charge separation and generates ROS on catalyst surfaces through friction, independent of light illumination [14,15,16,17]. This makes it particularly suitable for treating high-concentration, low-transparency wastewater. However, the development of tribocatalysis faces the following two major constraints: (i) its reliance on expensive synthetic catalysts (e.g., BaTiO_3_, TiO_2_), hindering large-scale application, and (ii) limited degradation efficiency for stubborn pollutants like Rhodamine B (RhB), which contains stable C–C and C–N bonds [18,19]. Currently, successful tribocatalytic degradation has been reported for 100 mg/L of Methyl Orange (MO), but only for 50 mg/L of RhB [20,21,22], leaving the efficient treatment of higher-concentration RhB largely unexplored. Moreover, natural CaCO_3_-based materials derived from shell waste have not yet been investigated as tribocatalysts.

To overcome these barriers, this study proposes a novel “waste-treats-waste” strategy by converting waste oyster shells into a low-cost, high-performance tribocatalyst. We prepared calcined natural shell particles (CNSP) via simple thermal treatment and employed them for the efficient degradation of high-concentration RhB solutions (50–300 mg/L). The catalytic performance under various conditions was systematically evaluated, and the underlying mechanism, particularly the generation of ROS, was thoroughly investigated. This work not only provides a sustainable pathway for shell waste valorization but also introduces an efficient, cost-effective, and green technology for recalcitrant organic wastewater treatment, contributing to dual resource cycles and environmental sustainability.

## 2. Materials and Methods

### 2.1. Materials and Their Characterization

This study used waste oyster shells as the raw material. The shells were washed repeatedly with deionized water to remove sand and organic residues and were then dried in an oven at 80 °C for 24 h. The shell powder was calcined in air using a muffle furnace (KSL-1700X, HF-Kejing Ltd., Hefei, China). It was heated to 800 °C at a ramp rate of 5 °C/min and then allowed to cool down directly without a holding period at the peak temperature. The resulting material was ground and sieved to obtain calcined oyster shell powder (CNSP). The high-purity (99.99 wt%) RhB powder used in this study was purchased from Damao Chemical Reagent Factory in Tianjin, China. The crystal structure of nanoparticles was characterized by X-ray diffraction (XRD) with Cu-K radiation (Philips X-ray diffractometer system, Philips, Almelo, The Netherlands). Their morphology was analyzed by field emission scanning electron microscopy (FESEM, JEOL JSM-6700F, JEOL Ltd., Tokyo, Japan).

### 2.2. RhB Degradation Experiment

The tribocatalytic degradation experiment was conducted. The catalytic performance was evaluated by degrading RhB. An amount of 0.3 g or 0.5 g of CNSP catalyst was dispersed in RhB solutions at concentrations of 50, 100, 200, and 300 mg/L, respectively, in 100 mL beakers. Using a homemade PTFE magnetic stirrer disk, the mixture was stirred at an average speed of 400 revolutions per minute on the H03-A model magnetic stirrer to provide continuous mechanical friction energy. The entire reaction system was placed in a dark chamber to eliminate photodegradation interference. Samples of 2 mL of solution were taken at predetermined time intervals and filtered through a 0.22 μm pore membrane to remove catalyst particles.

This study used a self-designed PTFE magnetic rotating disk as the core driving component for the tribocatalytic reaction. The disk base had a diameter of 35 mm and a thickness of 5 mm. A cross-shaped groove was machined on one main flat surface. The groove had a width of 4 mm and a depth of 2 mm. This sealed the magnets inside. The assembled rotor could be driven by a standard magnetic stirrer (H03-A, SH-Meiyingpu Ltd., Shanghai, China). The cross-shaped grooves on the disk surface helped catalytic particles enter the friction interface between the disk and the container bottom during stirring. This significantly enhanced mechanical energy transfer and catalytic efficiency. This PTFE magnetic rotating disk, designed specifically for tribocatalytic experiments, is referred to as the PTFE magnetic rotor (Supplementary Figure S1) in the following sections.

At the specified time point, three milliliters of the sample was collected each time and divided equally. Centrifugation at 8000 revolutions per minute for 5 min was performed to remove nanoparticles. Subsequently, the sample was analyzed using an ultraviolet-visible spectrophotometer (UV-2550; Shimadzu Corporation, Kyoto, Japan) within the wavelength range of 200–650 nanometers. The parameters for the ultraviolet-visible absorption spectrum were set as follows: scanning range 200–650 nanometers, wavelength step size 1.0 nanometer, slit width 2 nanometers. The degradation efficiency of the organic dye was calculated using the formula D = 1 − A/A_0_, where A_0_ and A represent the initial absorbance and residual absorbance at the characteristic peaks of the dye, respectively.

### 2.3. Detection of Active Species

In the detection of hydroxyl radicals, 10 mL of deionized water, 50 µL of 5,5-dimethyl-1-pyrroline-N-oxide (DMPO), and 0.15 g of shell powder were introduced into a glass beaker (φ 45 mm × 60 mm) with a glass bottom. DMPO was employed as a spin-trapping agent to capture transient hydroxyl radicals for EPR detection. For the detection of superoxide radicals, an identical experimental setup was used, except that 10 mL of methanol was substituted for deionized water, and DMPO was similarly used to trap superoxide radical species. Each beaker was equipped with a PTFE magnetic stirring disc, and the mixtures were stirred at 400 rpm for 15 min under dark conditions at ambient temperature. Subsequently, the generated radicals were characterized using an electron paramagnetic resonance (EPR) spectrometer (Model A300-10/12; Bruker Corporation, Berlin, Germany).

## 3. Results

### 3.1. Materials Information

**Figure 1:**
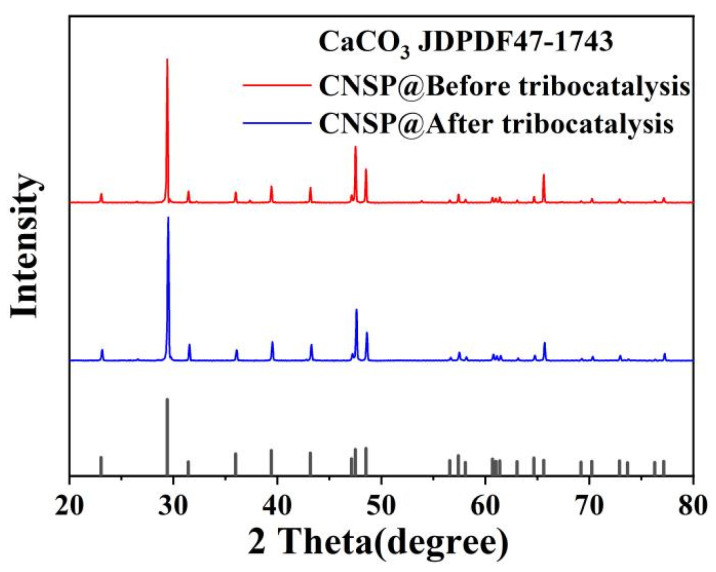
XRD patterns of CNSP particles before and after tribocatalysis.

Figure 1 shows XRD patterns of CNSP before and after tribocatalytic treatment. All observed diffraction peaks corresponded to the calcite structure of calcium carbonate (CaCO_3_) and matched PDF card number 47-1743. The XRD pattern after tribocatalytic treatment showed lower overall diffraction peak intensity compared to before tribocatalytic treatment, especially at the main diffraction peak positions of CaCO_3_. Although the peak intensities changed, all diffraction peaks clearly belonged to CaCO_3_. This indicated that the crystalline phase structure of CaCO_3_ remained stable during tribocatalytic reactions. No new phases were detected. Notably, the positions and shapes of the main diffraction peaks did not change significantly. Only slight intensity fluctuations occurred. This further confirmed that crystallinity remained unchanged. Additionally, the main diffraction peaks before and after tribocatalysis showed strong and sharp characteristics. This indicated high crystallinity of CNSP, which is crucial for effective tribocatalytic performance [23].

According to the XRD refinement results summarized in Table 1, obtained from multiple independent refinements, calcite-type CNSP maintained stable lattice parameters along the a-axis during tribocatalytic treatment, with the a-axis value remaining unchanged at 4.991 Å before and after reaction. In contrast, the c-axis lattice parameter exhibited only a slight variation, increasing marginally from 17.067 Å to 17.069 Å after tribocatalysis, while the unit cell volume showed no obvious change, remaining nearly constant at 368.2–368.3 Å^3^. These results indicate that the crystal structure of CNSP retains overall stability under mechanical stress, without significant lattice distortion during the tribocatalytic process. The high crystallinity and structural stability of CNSP are crucial for maintaining consistent surface properties and facilitating efficient charge transfer/accumulation during tribomechanical stress, which underpins its tribocatalytic activity. Specifically, the stable a-axis parameters preserved the integrity of the Ca-O covalent network. The slight expansion of the c-axis promoted interface strain-induced polarization. This process enhanced charge separation efficiency during the tribocatalytic reaction.

**Figure 2:**
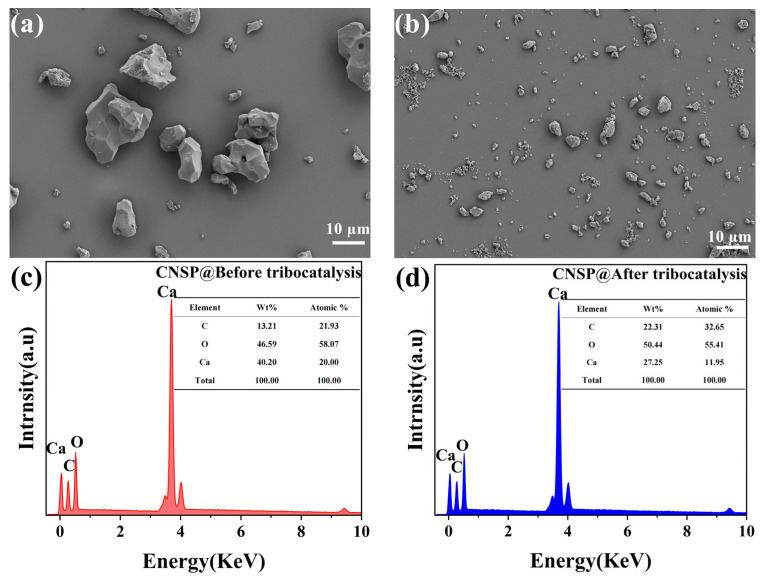
SEM images, EDX spectra, and particle size distributions of CNSP particles: (**a**) SEM before tribocatalysis; (**b**) SEM after; (**c**) EDX before; (**d**) EDX after.

Figure 2 shows SEM images comparing the morphology of CNSP before and after tribocatalysis. In Figure 2a, the particles are larger, with some forming agglomerates. In Figure 2b, the particles become smaller and are dispersed in the background. This indicates that the tribocatalytic process caused particle breakage or refinement. The distribution of particles in Figure 2a is uneven, with areas of large agglomerates. In contrast, Figure 2b shows a more uniform particle distribution. Similar particle refinement has been reported in ZnO-based tribocatalysts and was shown to enhance degradation kinetics by improving catalyst–reactant contact [23]. This uniformity after tribocatalysis helped ensure even reactions. It allowed better contact between reactants and the catalyst. It reduced uneven reactions caused by local particle accumulation. This improved the stability and efficiency of the reaction system. The particle surfaces in Figure 2a are rough with sharp edges. In Figure 2b, the surfaces are smoother. This change in surface morphology resulted from the modification of particle surfaces by physical wear during the tribocatalytic process. EDS analysis in Figure 2c,d shows that the main components of CNSP (Ca, O, etc.) did not change significantly after tribocatalytic reactions.

### 3.2. Tribocatalytic Degradation of RhB Solutions

RhB contains C–C and C–N bonds, making it more difficult to degrade than traditional dyes [18,19]. High-concentration RhB solutions are difficult to degrade. Few studies have reported the degradation of high-concentration RhB solutions. For RhB, tribocatalytic degradation was reported at 50 mg/L [20]. For MO, tribocatalytic degradation reached 100 mg/L [21]. No studies have focused on RhB degradation above 50 mg/L. Thus, we used high-concentration RhB solutions to study the performance of CNSP in dye degradation.

**Figure 3:**
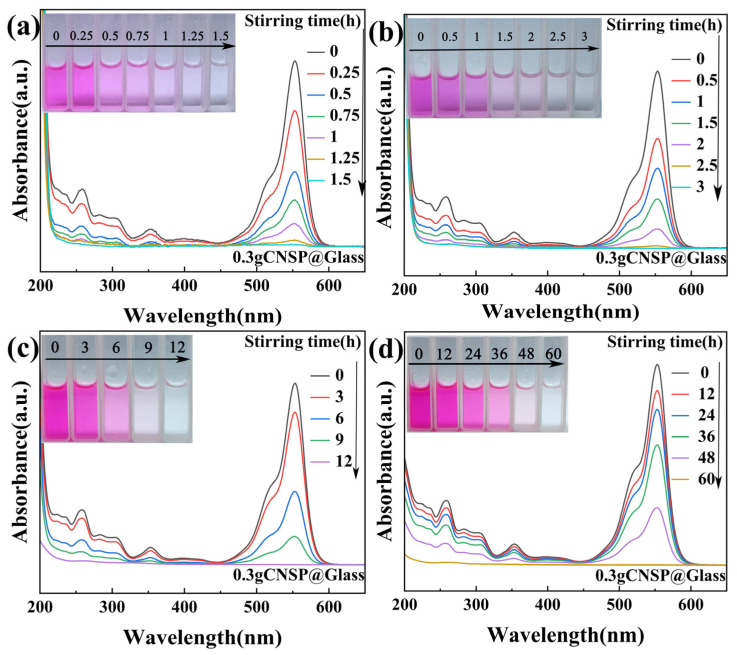
UV-Vis spectra of RhB solutions degraded by CNSP (0.3 g) at different concentrations: (**a**) 50; (**b**) 100; (**c**) 200; (**d**) 300 mg/L.

Figure 3 shows the degradation process of RhB at different concentrations using 0.3 g of CNSP as a catalyst. Remarkably, CNSP-mediated tribocatalysis achieved complete degradation of all high-concentration RhB solutions. However, the required time varied. Figure 3a shows that 50 mg/L of RhB solution degraded in 1.5 h. The degradation efficiency reached 99.3%. Figure 3b shows that 100 mg/L of RhB solution degraded in 3 h. The degradation efficiency reached 99.6%. Figure 3c shows that 200 mg/L of RhB solution degraded in 12 h. The degradation efficiency reached 99.9%. Figure 3d shows that even at 300 mg/L ultra-high concentration, a 99.3% degradation rate was achieved after 60 h.

The results show that, as RhB concentration increased, the time required for complete degradation by 0.3 g of CNSP also increased. However, the degradation efficiency at each concentration was better than reported in the literature [20,21,22]. UV-vis monitoring showed that the intensity of the characteristic absorption peak at 554 nm continuously decreased with magnetic stirring time. This continued until the solution was completely decolorized. Notably, all RhB characteristic peaks in the spectrum eventually disappeared. No new absorption peaks appeared. This indicates that RhB molecules were completely mineralized into CO_2_ and H_2_O.

**Figure 4:**
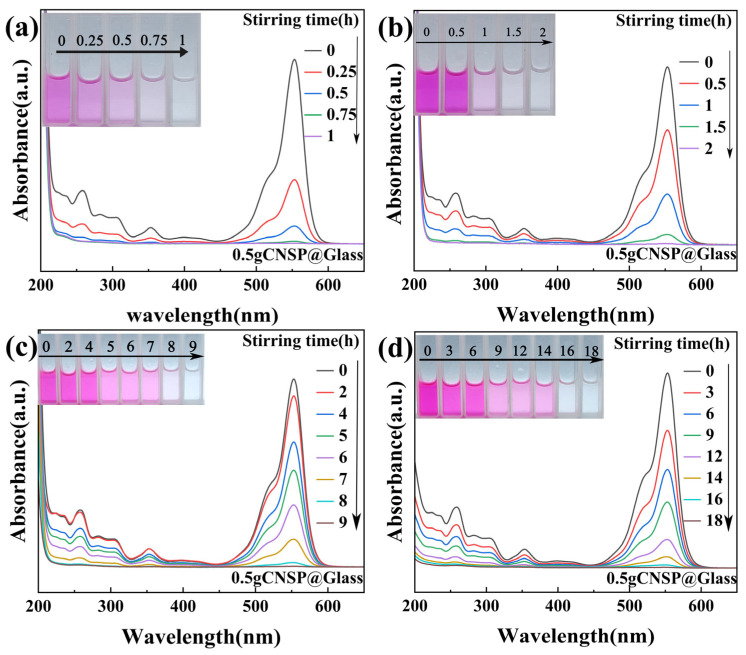
UV-Vis spectra of RhB solutions degraded by CNSP (0.5 g) at different concentrations: (**a**) 50; (**b**) 100; (**c**) 200; (**d**) 300 mg/L.

UV-Vis absorption spectroscopy monitored the degradation of different RhB concentrations by 0.5 g of CNSP in a glass-bottom beaker. This systematically evaluated catalyst dosage effects on tribocatalytic degradation performance. Figure 4 shows the degradation process with 0.5 g of CNSP. Figure 4a shows 50 mg/L of RhB degraded in 1 h. The degradation efficiency reached 99.7%. Figure 4b shows 100 mg/L of RhB degraded in 2 h with magnetic stirring. The degradation efficiency reached 99.8%. Figure 4c shows 200 mg/L of RhB degraded in 9 h with magnetic stirring. The degradation efficiency reached 99.7%. Figure 4d shows that, even at 300 mg/L ultra-high concentration, 99.8% degradation was achieved after 18 h.

**Figure 5:**
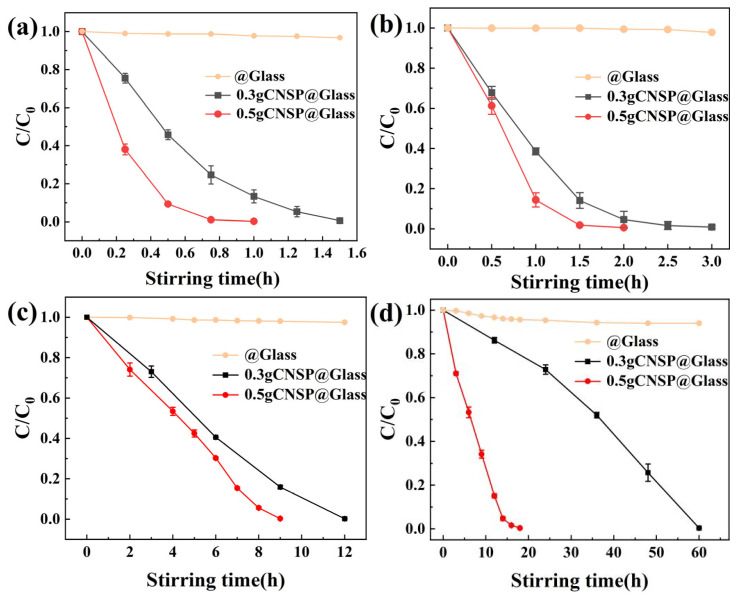
The relationship between the C/C_0_ ratio of different RhB solutions catalyzed by 0.5 g and 0.3 g of CNSP and magnetic stirring time in a glass beaker for different initial concentrations: (**a**) 50; (**b**) 100; (**c**) 200; (**d**) 300 mg/L.

Figure 5 compares the degradation curves of RhB solutions with different initial concentrations using 0.3 g and 0.5 g of CNSP catalyst in glass beakers. All experiments were performed independently at least three times, and the presented data represent the average values. Selected data points are shown with error bars corresponding to the standard deviation, demonstrating good experimental reproducibility. It should be noted that achieving complete degradation required extended durations (e.g., up to 60 h for 0.3 g and 18 h for 0.5 g at 300 mg/L). While the system demonstrates clear catalytic activity, practical application would benefit from further process intensification. Future studies could explore the effects of higher stirring speeds, alternative reactor geometries, or other operational modifications to potentially shorten the treatment time.

Results showed that the C/C_0_ ratio decreased steadily over time for both catalyst masses and all RhB concentrations. The ratio approached zero eventually, indicating efficient degradation of RhB solutions across the 50–300 mg/L concentration range. As a control, the blank experiment without CNSP showed almost no degradation. Notably, at the same RhB concentration, the degradation rate with 0.5 g of CNSP was significantly higher than that with 0.3 g. As RhB concentration increased, the time difference between the two masses became more pronounced. This pattern confirmed that increasing catalyst mass enhanced tribocatalytic efficiency. The literature generally reports a negative correlation between tribocatalytic degradation efficiency and dye initial concentration [24]. Our control experiment further showed that, without catalysts, 300 mg/L of RhB solution had only 6% degradation after 60 h of magnetic stirring. This result was nearly negligible. Based on these comparisons, the high degradation performance across a broad concentration range, as shown in Figure 5, significantly expanded the application boundaries of current tribocatalytic systems.

To investigate the cyclic stability of CNSP particles, a cyclic test was conducted in a glass-bottom beaker. In each cycle, 30 mL of RhB solution at concentrations from 50 to 300 mg/L was mixed with 0.50 g of CNSP particles in a glass-bottom beaker. The mixture was magnetically stirred using a self-made PTFE magnetic rotating disk for 1 h, 2 h, 9 h, or 18 h. A self-made PTFE magnetic stirrer disk was used to stir the mixture at 400 rpm to provide continuous mechanical friction energy. The entire reaction system was placed in a dark chamber to eliminate photodegradation interference. Samples of 2 mL of solution were taken at predetermined time intervals and filtered through a 0.22 μm pore membrane to remove catalyst particles. The supernatant was analyzed with a UV-Vis spectrophotometer. The CNSP particles were washed with deionized water and reused in the next cycle. The recovered catalysts were then dried at 75 degrees Celsius for 2 h, and the mass loss or content changes of the catalysts in each cycle were corrected and supplemented. Six consecutive cycles were completed for four different RhB concentrations. The degradation efficiency data from all cycles are summarized in Table 2. Results showed that CNSP particles maintained excellent cyclic stability under all concentration conditions. The degradation efficiency remained high after six consecutive cycles.

**Figure 6:**
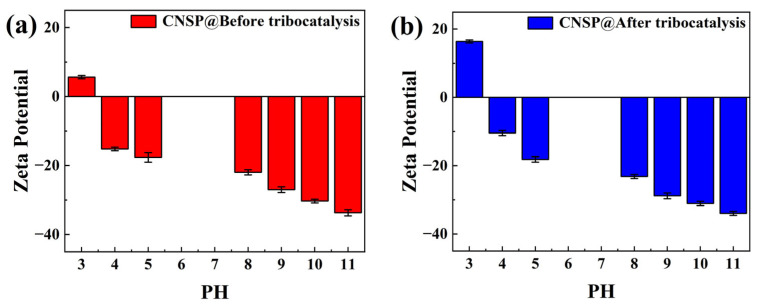
Zeta potentials of CNSP particles: (**a**) before tribocatalysis; (**b**) after tribocatalysis.

Figure 6a shows the zeta potential of CNSP particles before tribocatalytic treatment. Figure 6b shows the zeta potential change of CNSP particles after treatment. According to literature, the absolute value of the zeta potential can assess material stability at different pH values [25]. Compared to Figure 6a, the zeta potential of CNSP particles increased significantly at a pH of 3 after tribocatalytic treatment. It rose from 5.63 mV to 16.4 mV. The absolute value of the zeta potential also increased at other pH values. This indicates that the tribocatalytic process significantly enhanced the surface charge density of CNSP particles. This increased charge density improved electrostatic repulsion between particles. It reduced particle aggregation. It increased effective catalytic surface area. It improved colloidal stability. The increase in the absolute zeta potential value has important effects on catalyst performance. First, enhanced electrostatic repulsion maintains particle dispersion in solution. This increases the effective surface area of the catalyst. A larger effective surface area provides more active sites for reactions. This improves catalytic efficiency. Higher zeta potential values also enhance the electrostatic adsorption of charged pollutants. This promotes catalytic reactions.

**Figure 7:**
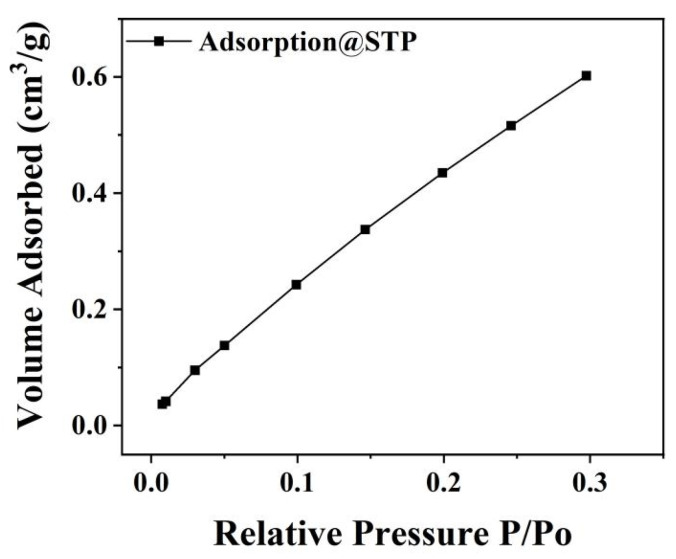
BET surface area data of CSNP particles.

Figure 7 shows the adsorption isotherm of CNSP at 200 °C. The BET analysis revealed a specific surface area of 2.6 m^2^/g. This indicates that CNSP has very low porosity and specific surface area. Specific surface area relates to adsorption capacity [26,27]. The low specific surface area of CNSP limits its adsorption ability, leading to weak adsorption. This structure explains that RhB degradation occurs through surface mechanical–chemical effects during tribocatalysis, not through the adsorption mechanism of traditional porous materials. This provides key evidence for understanding its tribocatalytic performance.

### 3.3. Mechanism Study on Tribocatalytic Degradation of RhB by CNSP Particles

**Figure 8:**
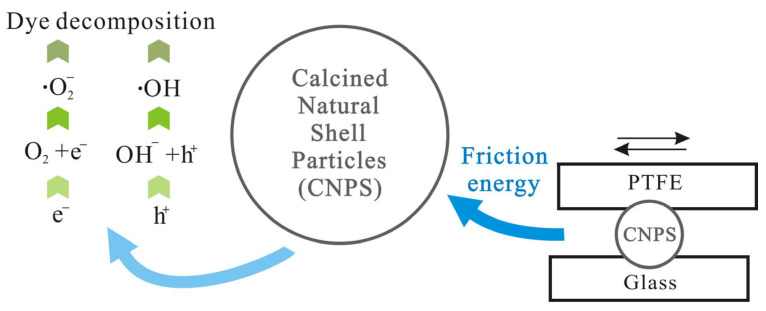
Schematic depiction of the tribocatalytic degradation of organic dyes by CNSP.

The outstanding tribocatalytic performance of CNSP in degrading high-concentration RhB is attributed to a surface activation mechanism driven by mechanical friction, rather than a conventional semiconductor-like electron-hole pair generation. The integrated evidence from zeta potential, EPR, BET, and SEM analyses supports a coherent mechanism centered on contact electrification-induced reactive oxygen species (ROS) generation. The tribocatalytic mechanism is shown in Figure 8.

**Figure 9:**
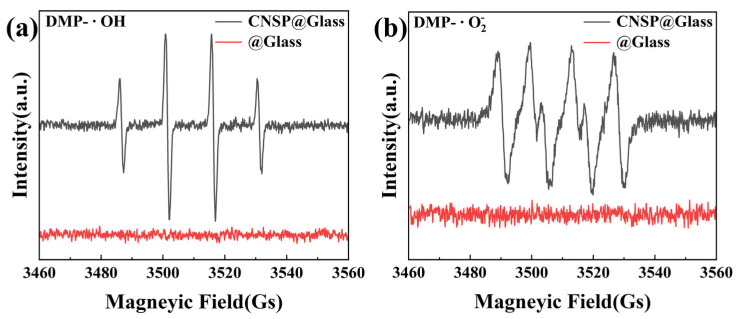
EPR spectra obtained for CNSP particles that were stirred using PTFE magnetic rotary disks in glass beakers containing (**a**) deionized water with DMPO as the spin-trapping agent and (**b**) methanol with DMPO as the spin-trapping agent.

EPR spectroscopy (9.85 GHz microwave frequency, 100 kHz modulation frequency) showed that CNSP particles generate radicals under magnetic stirring. After stirring CNSP particles in deionized water for 15 min, signals of hydroxyl radicals (•OH) and superoxide radicals ($•{O_{2}}^{-}$) were detected. Figure 9a shows the EPR spectrum of •OH in the CNSP@Glass system. A typical •OH signal was observed in the magnetic field range of 3460–3560 G. This signal appeared as a quadruple peak with an intensity ratio of 1:2:2:1. Hydroxyl radicals have high reactivity and can effectively attack and degrade organic pollutants [28,29]. Figure 9b shows the EPR spectrum of $•{O_{2}}^{-}$ in the same CNSP@Glass system. The characteristic signal of $•{O_{2}}^{-}$ was observed in the same magnetic field range. This signal appeared as four equal-intensity peaks with an intensity ratio of 1:1:1:1. These results indicate that superoxide radicals were successfully generated during the tribocatalytic process. Notably, under identical testing conditions, no discernible EPR signals of •OH or $•{O_{2}}^{-}$ were observed in the @Glass system without CNSP, indicating that radical generation originates from the presence of CNSP during the tribocatalytic process. As an important reactive oxygen species, superoxide radicals also participate in various oxidation reactions and promote the degradation of organic pollutants [30,31]. Overall, the EPR spectra in Figure 9a,b confirm that the tribocatalytic process in the CNSP@Glass system effectively generates •OH and $•{O_{2}}^{-}$. These active radicals are critical for degrading organic pollutants. The clear signals of the two key radicals show their generation. Their intensity indicates that the tribocatalytic process significantly enhances radical production efficiency. During the tribocatalytic process, •OH and $•{O_{2}}^{-}$ react with the chromophores of RhB. This causes structural breakdown and degradation of the dye. This process includes oxidation reactions. It also involves electron transfer and radical reactions. The dye is ultimately converted into harmless mineralized products. This mechanism provides a key molecular-level explanation for the tribocatalytic degradation of organic pollutants. The results further confirm the efficiency and feasibility of tribocatalytic systems in environmental remediation. They provide important evidence for understanding and optimizing the degradation mechanism of organic pollutants.

During vigorous magnetic stirring, continuous friction and collision occur between the CNSP particles and the inner wall of the glass beaker, as well as among the particles themselves. This mechanical interaction is likely to lead to contact electrification (triboelectric charging), causing charge transfer and accumulation on the surfaces of CNSP particles [14,16]. Direct evidence for this surface charge accumulation comes from the zeta potential measurements (Figure 6). After tribocatalysis, the surface charge density of CNSP increased significantly, with the zeta potential rising from +5.63 mV to +16.4 mV at a pH of 3. The enhanced positive surface potential strengthens the electrostatic interaction with anionic dye molecules and is likely to indicate a triboelectrically charged state that is crucial for subsequent redox reactions.

The accumulated surface charges on CNSP are likely to create a localized high-energy environment at the solid–liquid interface. These charges are likely to be directly injected into the surrounding aqueous medium or interact with adsorbed species, initiating interfacial electrochemical reactions [15,23]. Specifically, electrons are likely to reduce dissolved oxygen (O_2_) to generate superoxide anion radicals ($•{O_{2}}^{-}$), while the positively charged sites or local oxidative environments are likely to oxidize water or hydroxide ions to produce hydroxyl radicals (•OH). EPR spectroscopy provides definitive proof for this step. As shown in Figure 9, characteristic signals for both •OH (1:2:2:1 quartet) and $•{O_{2}}^{-}$ (1:1:1:1 quartet) were clearly detected in the CNSP suspension under magnetic stirring. The generation of these highly oxidative ROS is the pivotal step that drives the degradation of RhB.

The role of these radicals in pollutant decomposition is well-established. The •OH and $•{O_{2}}^{-}$ radicals are likely to non-selectively attack the chromophoric structure (e.g., the xanthene ring) and the ethylamine groups of RhB molecules through oxidation, cleavage of C–C and C–N bonds, and ring-opening reactions [32,33]. This process is likely to result in the stepwise decomposition of RhB into smaller organic intermediates and, ultimately, to complete decolorization into CO_2_, H_2_O, and inorganic ions, as evidenced by the gradual disappearance of all characteristic UV-Vis absorption peaks without the emergence of new ones (Figure 3 and Figure 4).

Two key auxiliary findings further refine and support this mechanism: (i) BET Analysis: The very low specific surface area of CNSP (2.635 m^2^/g, Figure 7) indicates minimal porosity and adsorption capacity. This effectively rules out physical adsorption as a primary removal pathway, confirming that the observed RhB removal is due to a surface-mediated tribochemical degradation process. (ii) SEM Analysis: The particle morphology changes after tribocatalysis (Figure 2)—from larger agglomerates to smaller, more dispersed particles with smoother surfaces—suggest particle refinement due to mechanical wear. This size reduction is likely to increase the effective contact area between CNSP and the reactor surface, thereby enhancing the efficiency of contact electrification and tribocatalytic activity.

In conclusion, the proposed mechanism for CNSP involves the following: (i) mechanical energy input through stirring causes friction and contact electrification; (ii) resulting surface charge accumulation (supported by zeta potential) drives interfacial electrochemical processes; (iii) these processes generate ROS (•OH and $•{O_{2}}^{-}$, confirmed by EPR), which are the primary agents for oxidizing and mineralizing RhB. This mechanism, leveraging waste-derived CaCO_3_, presents a novel and efficient pathway for tribocatalytic wastewater treatment, distinct from photocatalysis or adsorption-based methods. A conceptual illustration of this process is provided in Figure 8.

Despite the development of various techniques for RhB solution degradation, mainstream methods such as photocatalysis face significant challenges when pollutant concentrations exceed 50 mg/L. The performance improvement of existing photocatalytic technologies typically relies on catalyst modification and optimization. However, these improvements involve high costs. They also lead to a sharp decline in photocatalytic efficiency as pollutant concentration increases. By contrast, CNSP as a tribocatalyst catalyst shows unique advantages. This natural material efficiently degrades RhB solutions across a wide concentration range of 50–300 mg/L. It generates hydroxyl radicals (•OH) and superoxide radicals ($•{O_{2}}^{-}$) through mechanical energy. This enables the rapid oxidative mineralization of pollutants. Additionally, CNSP has a low cost and is environmentally friendly. It maintains stable catalytic activity after six consecutive cycles. This proves its long-term reliability and reusability in practical applications. Overall, these results indicate that tribocatalysis represents a complementary strategy to traditional photocatalysis, effectively addressing the challenges associated with high-concentration and low-transparency organic wastewater and offering a sustainable pathway for the treatment of refractory organic pollutants.

## 4. Conclusions

In summary, this study successfully developed a low-cost and eco-friendly tribocatalyst (CNSP) from waste oyster shells for the efficient degradation of high-concentration RhB dye wastewater. CNSP exhibited remarkable adaptability and activity, completely degrading RhB across an extensive concentration range (50–300 mg/L). Evidence indicates that friction-induced charge excitation likely facilitates the robust generation of key reactive oxygen species (•OH and $•{O_{2}}^{-}$), as confirmed by EPR spectroscopy. Beyond introducing a high-performance tribocatalyst, this work proposes an innovative “waste-treats-waste” paradigm, the first reported tribocatalytic degradation of RhB above 50 mg/L, using a natural shell-derived material, transforming an environmental burden (waste shells) into a functional material for pollution remediation and reducing landfill burden, thereby promoting resource sustainability. Although the laboratory-scale results are promising, future work should focus on scaling up the process, testing CNSP’s efficacy in real industrial effluents containing mixed pollutants, exploring systematically reactor design and operating parameter optimization, and further elucidating the detailed surface charge transfer mechanisms to guide the design of next-generation tribocatalysts.

## Figures and Tables

**Table 1:** Rietveld refined structural parameters of CNSP particles before and after tribocatalysis ceramics.

Sample	a	c	a/c	V
CNSP before tribocatalysis	4.991	17.067	0.2925	368.2
CNSP after tribocatalysis	4.991	17.069	0.2924	368.3

**Table 2:** Cyclic stability of 0.5 g of CNSP particles: tribocatalytic degradation rates of RhB solutions at concentrations from 50 to 300 mg/L after magnetic stirring for 1 h, 2 h, 9 h, and 18 h in a glass-bottom beaker.

	Cycle Number					
Degradation Rate @Glass	1	2	3	4	5	6
50 mg/LRhB	0.997	0.997	0.996	0.993	0.988	0.958
100 mg/LRhB	0.998	0.997	0.997	0.997	0.993	0.969
200 mg/LRhB	0.988	0.997	0.994	0.992	0.969	0.952
300 mg/LRhB	0.998	0.996	0.996	0.992	0.992	0.987

## Data Availability

The original contributions of this study are contained within the article, and any further inquiries may be addressed to the corresponding authors.

## Supplementary Materials

The following supporting information can be downloaded at: https://www.mdpi.com/article/10.3390/nano16030194/s1, Figure S1: Photographs of home-made PTFE magnetic rotary disk: (a) front surface of cross-shaped grooves; (b) front surface of top cover; (c) side surface.

## References

[1] Giordano L., Ferraro L., Caroppo C., Rubino F., Buonocunto F., Maddalena P. (2022). A method for bivalve shells characterization by FT-IR photoacoustic spectroscopy as a tool for environmental studies. MethodsX.

[2] Asmare Z.G., Aragaw B.A., Atlabachew M., Dubale A.A., Mohammed K.S. (2025). Facile Preparation of a Kaolin-Supported CuO Catalyst and Investigation of Its Catalytic Performance for Methylene Blue Dye Degradation in Aqueous Solution. ACS Omega.

[3] Kumaravel S., Kim H. (2023). Development and characterization of solar active Ag-ZnO/g-C_3_N_4_ as a highly efficient photocatalyst for the detoxification of organic pollutant. Colloids Surf. A Physicochem. Eng. Asp..

[4] Moradi R., Mehdizade S. (2023). Adsorption of Acid Orange 7 Dye Pollutant from Aqueous Solutions Using Modified Multi-Walled Carbon Nanotubes. Russ. J. Phys. Chem. A.

[5] Yan J., Zhou T., Peng J., Wang H., Jiang L., Cheng Q. (2024). Sustainable liquid metal-induced conductive nacre. Sci. Bull..

[6] Meng L., Liu L., Zhou Q., Shen J., Li J., Meng L. (2025). Effect of Modified Shellac on Mechanical Properties of Reed Fiber/Polyethylene Composites. China For. Prod. Ind..

[7] Cahyati N., Sari M., Yusuf Y. (2024). Properties of Carbonated Hydroxyapatite-Based Scaffold from Oyster Shells Composited with Honeycomb and Polyethylene Oxide for Bone Tissue Engineering Applications. Key Eng. Mater..

[8] Ji L., Song W., Wang Y., Guo J., Wang Y., Cai L. (2017). Hermodynamics of the Adsorption of Cadmium (II) and Lead (II) Ions inCalcined Purple Mussel Shells. Mod. Food Sci. Technol..

[9] Ismail R., Cionita T., Lai Y.L., Fitriyana D.F., Siregar J.P., Bayuseno A.P., Nugraha F.W., Muhamadin R.C., Irawan A.P., Hadi A.E. (2022). Characterization of PLA/PCL/Green Mussel Shells Hydroxyapatite (HA) Biocomposites Prepared by Chemical Blending Methods. Materials.

[10] Kobanoğlu M.H., Çoruh S., Gürkan E.H. (2025). Utilizing seashells for the removal of malachite green dye pollution: A sustainable approach in textile wastewater treatment. Fuller. Nanotub. Carbon Nanostructures.

[11] Hieu N.H., Dat T.D., An H., Nam N.T.H., Khoa T.D., Vu N.H., Minh D.T.C. (2025). Co-doping g-C3N4 with tungsten and phosphorus for efficient photofixation of nitrogen to ammonia, degradation of organic pollutants, Cr (VI) reduction, and antibacterial activity. Inorg. Chem. Commun..

[12] Fan C., Li M., Zhang X., Tian N. (2025). Mo vacancy engineered Bi_2_MoO_6_ nanosheets for efficient photocatalytic pollutant degradation. Mater. Res. Bull..

[13] Zou M., Wu J., Chen R., Zhao H., Zhou H., Deng G., Zhou S. (2025). Preparation and photocatalytic properties of TiO_2_ based on burst-mode femtosecond laser and anodization. Opt. Mater..

[14] Cui X., Li P., Lei H., Tu C., Wang D., Wang Z., Chen W. (2022). Greatly enhanced tribocatalytic degradation of organic pollutants by TiO_2_ nanoparticles through efficiently harvesting mechanical energy. Sep. Purif. Technol..

[15] Li P., Tang C., Xiao X., Jia Y., Chen W. (2021). Flammable gases produced by TiO_2_ nanoparticles under magnetic stirring in water. Friction.

[16] Li P., Wu J., Wu Z., Jia Y., Ma J., Chen W., Zhang L., Yang J., Liu Y. (2019). Strong tribocatalytic dye decomposition through utilizing triboelectric energy of barium strontium titanate nanoparticles. Nano Energy.

[17] Ke S., Mao C., Luo R., Zhou Z., Hu Y., Zhao W., Chen W. (2024). Surprising Effects of Al_2_O_3_ Coating on Tribocatalytic Degradation of Organic Dyes by CdS Nanoparticles. Coatings.

[18] Wang D., Zhu Y., Li J., Yang W., Zhao Y., Chen G. (2025). Highly efficient photocatalytic degradation of organic pollutants by Z-scheme Ni-C_3_N_4_/P-C_3_N_4_ heterojunction. Colloids Surf. A Physicochem. Eng. Asp..

[19] Doumbia M., Guenfoud F., Boudriche L., Giannakis S. (2024). UVA-assisted photocatalytic removal of rhodamine B using green-synthesized activated carbon derived from date pits and impregnated with Ag/Ag_3_PO_4_. J. Coord. Chem..

[20] Zhu M., Song J., Ke S., Gu Y., Bing L., Shen Z., Chen W. (2025). Ti Coating-Enhanced Tribocatalytic Degradation of Organic Dyes by CdS Nanoparticles. Inorganics.

[21] Zhu M., Zhou Z., Gu Y., Bing L., Xie Y., Shen Z., Chen W. (2025). Powerful tribocatalytic degradation of methyl orange solutions with concentration as high as 100 mg/L by BaTiO_3_ nanoparticles. Nanomaterials.

[22] Mao C., Zhang Y.-C., Lei H., Jia X., Chen F., Yao W., Liu P., Chen W. (2024). Boosting tribo-catalytic degradation of organic pollutants by BaTiO_3_ nanoparticles through metallic coatings. Appl. Surf. Sci..

[23] Lei H., Cui X., Jia X., Qi J., Wang Z., Chen W. (2022). Enhanced Tribocatalytic Degradation of Organic Pollutants by ZnO Nanoparticles of High Crystallinity. Nanomaterials.

[24] Emmanuel S.S., Adesibikan A.A. (2024). Harvesting surface (interfacial) energy for tribocatalytic degradation of hazardous dye pollutants using nanostructured materials: A review. J. Chin. Chem. Soc..

[25] Saah F.K., Nagpal G. (2025). Selective sequential extraction of copper from soil using GSCQD/ZnFe_2_O_4_ nanocomposite in continuous flow system. Nano-Struct. Nano-Objects.

[26] Gupta V.K., Suhas (2008). Application of low-cost adsorbents for dye removal—A review. J. Environ. Manag..

[27] Chong J., Tai B., Zhang Y. (2024). Tribocatalysis effect based on ZnO with various specific surface areas for dye degradation. Chem. Phys. Lett..

[28] Che J., Gao Y., Hu Y., Song J., Zhang Y., Wu Z., Jia Q., Ma J., Jia Y. (2025). Efficient nano-Er_2_O_3_ tribocatalyst for mechanical friction-driven water purification. Ceram. Int..

[29] Cui X., Guo Z., Lei H., Jia X., Mao C., Ruan L., Zhou X., Wang Z., Chen F., Chen W. (2023). Tribo-Catalytic Degradation of Methyl Orange Solutions Enhanced by Silicon Single Crystals. Coatings.

[30] Mosleh A.T., Yousef T.A., Khairy M., Ferjani H., Almuhana A.R.Y., Zahran H.Y., Rahman A.E., Essam O.A., Abdelnasser M.I., Abdelbaset S.A. (2025). Multifunctional prospects of PMMA/Fe_2_O_3_@NiO nanocomposite membranes: Advanced optical, dielectric, and photocatalytic properties for electronic optoelectronic devices, and environmental applications. J. Sol-Gel Sci. Technol..

[31] de Oliveira M.F., de Freitas Filho R.L., Lago R.M., de Texeira Carvalho A.P., Ruiz Y.L., Aguilera L., da Silva R.S., Sampaio D.V., Vega N.C., Marin-Ramirez O. (2025). Single phase CaTiO_3_ nanoparticles by modified Pechini method: Structural, Rietveld refinement, optical and photocatalytic properties. Surf. Interfaces.

[32] Qasim M., Rizvi A.A., Rashid H., Li X., Alzahrani H.A.H., Althomali R.H., Alghamdi M.M., El-Zahhar A.A., Solre G.F.B., Asif S.U. (2025). High-efficiency RhB dye degradation using β-FeOOH nanorods via tribocatalysis. RSC Adv..

[33] Akram N., Guo J., Ma W., Guo Y., Hassan A., Wang J. (2020). Synergistic Catalysis of Co(OH)_2_CuO for the Degradation of Organic Pollutant Under Visible Light Irradiation. Sci. Rep..

