# Supplementary material for: Strong Tribocatalytic Degradation of Organic Pollutants by Natural Shell Particles
Source: Nanomaterials (Basel). 2026 Jan 30;16(3):194. doi: 10.3390/nano16030194 (PMC12899633; doi:10.3390/nano16030194)
Supplement: Supplementary file 1 [file nanomaterials-16-00194-s001.zip › nanomaterials-4094085-supplementary.pdf]

Supplementary Information

Strong Tribocatalytic Degradation of Organic Pollutants by Natural Shell Particles

Yuqin Xie [1], Mingzhang Zhu [1], Zhenming Xu [1], Lina Bing [1], Wanping Chen [2,*] and Zhenjiang Shen [1,*]

This study used a self-designed PTFE magnetic rotating disk as the core driving component for the tribocatalytic reaction. The disk base had a diameter of 35 mm and a thickness of 5 mm. A cross-shaped groove was machined on one main flat surface. The groove had a width of 4 mm and a depth of 2 mm. A PTFE cover plate with a thickness of 7 mm was also designed. A central cavity was machined on one side of the cover. Four commercial cylindrical neodymium-iron-boron magnets were precisely embedded in this cavity. Two magnets had dimensions of Φ5 mm × 25 mm, and the other two had Φ5 mm × 20 mm. The disk base and cover were firmly bonded using high-strength 502 adhesive. This sealed the magnets inside. The assembled rotor could be driven by a standard magnetic stirrer. The cross-shaped grooves on the disk surface helped catalytic particles enter the friction interface between the disk and the container bottom during stirring. This significantly enhanced mechanical energy transfer and catalytic efficiency. This PTFE magnetic rotating disk, designed specifically for tribocatalytic experiments, is referred to as the PTFE magnetic rotor in the following sections.

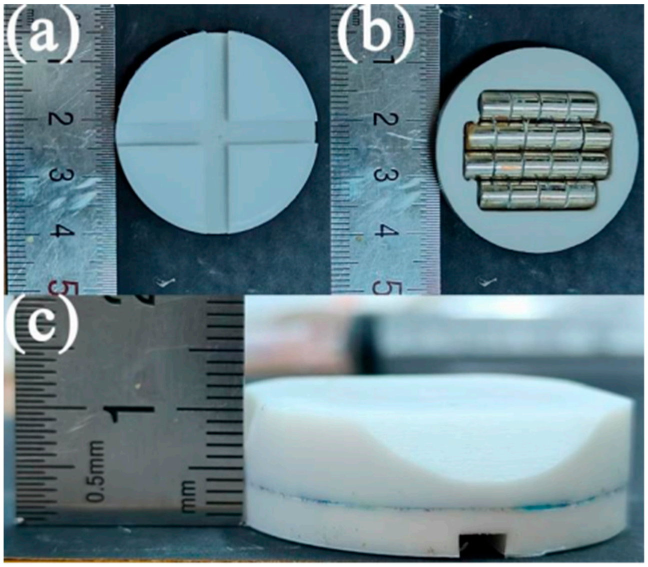

Supplementary Figure S1. Photographs of home-made PTFE magnetic rotary disk: (**a**) front surface of cross-shaped grooves; (**b**) front surface of top cover; (**c**) side surface.
